# Validation of the specific loss of interest and pleasure scale in non-clinical and clinical populations

**DOI:** 10.3389/fpsyg.2026.1706503

**Published:** 2026-03-24

**Authors:** Yujia Xu, Wenxia Liu, Li Liu, Chenglei Wang, Ting Li, Chao Yan, Xinhua Yang

**Affiliations:** 1 Shanghai Changning Mental Health Center, Affiliated Mental Health Center of East China Normal University, Shanghai, China; 2 School of Psychology and Cognitive Science, East China Normal University, Shanghai, China

**Keywords:** anhedonia, Chinese, recent changes, reliability, validation

## Abstract

**Introduction:**

The Specific Loss of Interest and Pleasure Scale (SLIPS) is a novel measure specifically designed to capture recent changes in anhedonia. The present study aimed to investigate its psychometric properties in Chinese settings.

**Materials:**

Participants included a non-clinical sample (*n* = 1,251) and a clinical sample (*n* = 224; 190 mixed psychiatric patients and 34 healthy controls). The exploratory factor analysis (EFA) using polychoric correlations with parallel analysis and the minimum average partial test, confirmatory factor analysis (CFA) using the WLSMV estimator, correlation analyses, and hierarchical regression models were used.

**Results:**

The unidimensional structure was supported. The CFA results in the non-clinical sample showed satisfactory model fit (*χ*^2^(227) = 375.771 (*p* < 0.001), scaled CFI = 0.981, scaled TLI = 0.979, scaled RMSEA = 0.032[90% CI: 0.026, 0.038], SRMR = 0.055). Internal consistency was robust (non-clinical: Cronbach’s *α* = 0.90[95%CI: 0.90, 0.91], McDonald’s *ω* = 0.91[95%CI: 0.90, 0.92]; clinical: Cronbach’s α = 0.96[95%CI: 0.95, 0.97], McDonald’s ω = 0.96[95%CI: 0.95, 0.97]). Test–retest reliability for a 1-month interval (*n* = 321) indicated moderate stability (ICC[2,1] = 0.55[95%CI: 0.47, 0.62]), and strict longitudinal measurement invariance was established (ΔCFI<0.01).

**Discussion:**

Validity analyses showed that the SLIPS correlated with state, trait, and social anhedonia but was distinct from guilt. Crucially, the SLIPS demonstrated incremental validity in predicting depressive symptoms and suicidal ideation compared with state and trait anhedonia. The Chinese version of the SLIPS demonstrated promising psychometric evidence in assessing changes in anhedonia. However, inferences regarding specific clinical subgroups (e.g., MDD) are limited by sample size and require further validation.

## 1 Introduction

Anhedonia is known as a heterogeneous symptom across psychiatric disorders, varying depending on which components of the pleasure networks are most affected (Winer et al., 2019). Traditionally, anhedonia has been assessed primarily as either a trait or a state construct (Loas et al., 2009). However, according to clinical diagnostic criteria, anhedonia is defined as a decrease in the ability to experience pleasure during the 2-week period, indicating a change from previous levels of functioning (American Psychiatric Association, 2013). This emphasis is potentially vital for understanding depressive symptoms and suicidal ideation as recent decreases in anhedonia may provide more valuable information that is not otherwise captured by traditional trait or state assessments.

Anhedonia is a multi-faceted construct, including trait, state, and symptom-based recent changes from baseline. Trait anhedonia, particularly in physical and social domains, has traditionally been assessed using the Revised Chapman Physical Anhedonia Scale and the Revised Chapman Social Anhedonia Scale (Chapman et al., 1976). However, these measures have been criticized as culturally biased (Rizvi et al., 2016), prompting the development of new trait-anhedonia measures. The Temporal Experience of Pleasure Scale differentiates between trait anticipatory and consummatory anhedonia (Gard et al., 2006), while the Anticipatory and Consummatory Interpersonal Pleasure Scale focuses on the social domain (Gooding and Pflum, 2014). Despite these advances, trait measures primarily evaluate an individual’s general ability to experience pleasure without a temporal component. Such static measures fail to align with the depressive diagnostic criteria that anhedonia is a diminished capacity to derive pleasure (Winer et al., 2019), limiting their clinical utility. State anhedonia, in contrast, has typically been assessed with the Snaith–Hamilton Pleasure Scale (Snaith et al., 1995), which measures consummatory hedonic tone over the previous several days. More recently, the Dimensional Anhedonia Rating Scale was developed to assess four components of anhedonia (interest, motivation, effort, and pleasure) across multiple reward domains (Rizvi et al., 2015). However, these state measures generally rely on retrospective pleasurable experiences (e.g., past 2 weeks) without comparing current emotional states with previous ones (Winer et al., 2019). Overall, while both trait- and state-based measures provide important perspectives on anhedonia, neither of these measures adequately capture short-term fluctuations or changes in this condition.

In fact, increasing evidence have suggested that recent changes in anhedonia are clinically important. The anhedonia subscale of the Beck Depression Inventory, indicating recent changes in loss of interest and pleasure, has been found to be specifically predictive of depression (Joiner et al., 2003). Among undergraduate students, recent changes in anhedonia were associated with current suicidal ideation, even after accounting for other depressive symptoms, as well as state and trait anhedonia (Winer et al., 2016; Yang et al., 2022). For psychiatric patients, only recent changes in anhedonia, rather than lifelong anhedonia, along with cognitive or affective symptoms of depression have emerged as the main risk factor for current suicidal ideation (Dosogne et al., 2022; Winer et al., 2014a; Winer et al., 2014b). Moreover, contemporary diagnostic criteria conceptualize anhedonia as a marked loss of interest, rather than simply a low absolute level of enjoyment. Thus, focusing on recent changes in anhedonia may be particularly useful for tracking short-term fluctuations in symptoms. This approach could help researchers and clinicians more effectively identify depression and the risk of suicidality (Winer et al., 2014a).

In contrast to measures that assess longstanding trait or current state levels of anhedonia, the Specific Loss of Interest and Pleasure Scale (SLIPS) was developed to specifically capture perceived recent changes in anhedonia (Winer et al., 2014b). The SLIPS focuses on experiences in the last 2 weeks and asks about current interest or pleasure relative to their usual level of functioning across different time periods and various activities. By explicitly anchoring each item to comparisons with the past, the scale is designed to detect recent declines in interest or enjoyment rather than static severity levels, thereby offering an important complement to existing level-based measures. The novel scale consists of 23 self-report items rated on a 4-point scale (0–3) and measures changes in the experience of, or interest in, previously pleasurable activities. For example, when responding to the item “Going out with friends,” the statement “I still enjoy it” reflects no change in interest or pleasure; “I do not enjoy it as much as I used to” reflects a modest decline; and “I no longer enjoy it” reflects a substantial decline. In contrast, “I have never enjoyed it” indicates that the activity has historically not been pleasurable and thus does not represent a recent change. In this way, the SLIPS differentiates between enduring low pleasure and recent loss of pleasure. Previous studies have demonstrated satisfactory psychometric properties for the English, Persian, and Israeli versions of the SLIPS (Daghigh et al., 2019; Ritsner and Ratner, 2019; Winer et al., 2014b). However, test–retest reliability, measurement invariance over time, and stability under negative social events of the SLIPS were not fully examined in these studies, and the applicability of the scale to non-Western samples remains unknown.

The purpose of the present study was to validate the translated version of the SLIPS in Chinese settings. We examined its factor structure, internal consistency, test–retest reliability, and convergent, divergent, and incremental validity in both non-clinical and clinical samples. Based on the previous research, we hypothesize that (a) the Chinese SLIPS would demonstrate a unidimensional factor structure consistent with the original scale; (b) the scale would show good internal consistency and test–retest reliability; (c) SLIPS scores would correlate positively with other measures of anhedonia and depressive symptoms while showing weaker associations with unrelated constructs; and (d) the SLIPS would exhibit incremental validity in predicting depression and suicidal ideation independent of level-based anhedonia measures. Since our data were collected from January to April 2020 during the coronavirus disease (COVID-19) outbreak, the impact of the lockdown on the SLIPS was also assessed. Although this was not part of the original aim of our study, the pandemic allowed us to evaluate the longitudinal measurement invariance of the SLIPS during lockdown and its predictive validity for depression and suicidal ideation.

## 2 Materials and methods

### 2.1 Participants

For the non-clinical sample, a total of 1,620 individuals from Hunan Province during January 2020 and April 2020, using a convenience sampling strategy, were initially approached. Of these, 1,468 agreed to participate and completed the survey, resulting in a response rate of 90.6%. Unexpectedly, the Wuhan lockdown began on 23 January 2020, due to the COVID-19 outbreak. Participants were again invited to complete the pandemic effect on mental health survey via email. Follow-up was completed between 14 February and 8 April 2020, during which China experienced a rapid increase in the number of COVID-19 cases and associated deaths.

The exclusion criteria were defined as follows: (1) repeated responses [participants who answered twice had their second answers removed (*n* = 123)], (2) completion time outliers, with completion time below the 2.5th percentile (187.60s) or above 97.5th percentile (3154.40s) (*n* = 81), and (3) incomplete surveys with missing data (*n* = 13). Finally, a total of 1,251 valid baseline responses were retained. The response rate was 85.2%. According to the pandemic outbreak, the participants were divided into a pre-lockdown sample (6–22 January 2020; *n* = 326) and a during-lockdown sample (23 January–9 March 2020; *n* = 925). Sociodemographic information is presented in Table 1. Among the pre-lockdown participants, 321 individuals completed the follow-up assessment during lockdown (14 February–8 April; drop-out rate = 1.5%; see Supplementary Table 1).

**Table 1:** Characteristics of participants.

Variables	Non-clinical sample (*n* = 1,251)				Clinical sample (*n* = 224)			
	Pre-lockdown (*n* = 326)	During-lockdown (*n* = 925)	*z/χ^2^*	*p*	Healthy control (*n* = 34)	Psychiatric patients (*n* = 190)	*z/χ^2^*	*p*
Age (median [Q1, Q3])	22.00 [20.00, 23.00]	21.00 [20.00, 22.00]	−7.29	<0.001	45.76 ± 9.60	48.46 ± 16.68	−1.01	0.310
Gender (female, *n* [%])	214 [65.6%]	691 [74.7%]	9.89	0.002	21 (61.8%)	120 (63.2%)	0.02	0.877
Education	15.09 ± 2.15	15.29 ± 2.07	−1.42	0.157	10.94 ± 3.12	10.88 ± 3.29	0.27	0.789
SLIPS	6.62 ± 6.32	7.21 ± 7.40	−0.52	0.607	8.12 ± 8.64	7.37 ± 10.13	1.15	0.251
SHAPS	23.25 ± 5.71	23.39 ± 6.09	0.31	0.758	21.29 ± 6.01	23.88 ± 5.88	−2.27	0.023
TEPS-ANT	37.34 ± 7.18	37.84 ± 6.96	−1.15	0.249	35.35 ± 7.56	33.41 ± 9.40	0.92	0.359
TEPS-CON	43.61 ± 8.39	44.27 ± 7.96	−1.44	0.150	42.79 ± 6.23	40.09 ± 10.76	1.43	0.152
ACIPS	76.51 ± 12.90	75.00 ± 13.73	1.81	0.070	77.68 ± 16.73	69.24 ± 18.18	2.23	0.026
SI	1.00 ± 1.71	1.22 ± 2.02	−1.17	0.245	0.65 ± 1.37	1.94 ± 2.37	−3.44	<0.001
BDI	7.63 ± 7.43	8.58 ± 8.58	−1.10	0.271	3.94 ± 4.53	8.75 ± 9.50	−2.33	0.020
Guilt	17.98 ± 5.32	18.66 ± 5.00	−1.94	0.053	-	-	-	-

SLIPS = Specific Loss of Interest and Pleasure Scale; SHAPS = Snaith-Hamilton Pleasure Scale; TEPS-ANT = Anticipatory anhedonia in Temporal Experience of Pleasure Scale; TEPS-CON = Consummatory anhedonia in Temporal Experience of Pleasure Scale; ACIPS = The Anticipatory and Consummatory Interpersonal Scale; BDI = 17 items of Beck Depression Inventory; SI = Suicidal Ideation; Data are presented as M ± SD.

For the clinical sample, 190 psychiatric patients (151 with chronic schizophrenia, 35 with remitted bipolar disorder, 4 with depression) and 34 healthy controls were recruited from Shanghai Changning Mental Health Center. This resulted in an unbalanced diagnostic distribution with a relatively small depression subsample, which we acknowledge as a limitation. The inclusion criteria for patients were as follows: (1) age ≥ 18 years old and (2) meeting DSM-IV diagnostic criteria based on SCID-I administered by an experienced psychiatrist. The exclusion criteria included the following: (1) patients with any other concurrent axis I disorders, (2) severe neurological disease, or (3) cognitive impairment that prevented the completion of the valid questionnaire. In the healthy control group, participants received a structured screening conducted by an experienced psychiatrist to ensure the absence of any neuropsychiatric symptoms (see Table 1).

### 2.2 Measures

#### 2.2.1 The Chinese version of the SLIPS

The original English version of the SLIPS contained 23 items that assess changes in anhedonic symptoms in response to social experiences within the past 2 weeks (Winer et al., 2014b). The scale is scored on a 4-point scale (0–3), with a higher score reflecting a greater severity of anhedonia reduction in interest or pleasure. For each item, response options indicate the degree of change in pleasure for a specific experience, ranging from not at all (0) to dramatic reduction (2) relative to the past. The fourth option (3; e.g., “I have never enjoyed this activity”) was recoded to 0 because it reflects a stable trait-like lack of enjoyment rather than a recent change. This scoring method yields a possible total score range of 0–46 and typically results in a positively skewed distribution, with the majority of participants endorsing few recent declines.

Approval was obtained from the original author of the scale for conducting the validation of the Chinese version. The current study followed guidelines for cross-cultural adaptation of self-report measures. The translation process involved two stages. The senior author and two psychiatrists first translated the SLIPS into Chinese. The translated version was then back-translated into English by two PhD students in psychology who were not involved in the original validation. With the English version confirmed by the original author, the final Chinese version was reviewed by an expert panel consisting of clinicians and researchers to ensure conceptual equivalence, cultural relevance, and comprehensibility for Chinese participants. The full set of the Chinese SLIPS items is provided in Appendix A.

#### 2.2.2 State anhedonia

The Chinese version of the Snaith-Hamilton Pleasure Scale (SHAPS) is a 14-item questionnaire with self-statements of pleasure in response to general pleasant experiences (Liu et al., 2012). Items are rated on a 4-point Likert scale from “totally false for me” to “totally true for me,” yielding a total score ranging from 0 to 14, with higher scores indicating greater state anhedonia. The Cronbach’s α for SHAPS in the current sample was 0.92 (95% CI [0.91, 0.92]), and McDonald’s ω was 0.92 (95% CI [0.91, 0.93]).

#### 2.2.3 Trait anticipatory and consummatory anhedonia

The Temporal Experience of Pleasure Scale (TEPS) was used to measure trait anticipatory and consummatory experiences of pleasure (Chan et al., 2012). It includes 20 items that capture four factors: the anticipatory pleasure subscale (TEPS-ANT) and the consummatory subscale (TEPS-CON). Items are rated on a 6-point Likert scale rated from “very false for me” to “very true for me,” producing a total score range of 20–120. Lower scores indicate greater anhedonia. The Cronbach’s α for the TEPS in the present sample was 0.87 (95% CI [0.86, 0.88]), and McDonald’s ω was 0.88 (95% CI [0.87, 0.89]).

#### 2.2.4 Trait social anhedonia

The Anticipatory and Consummatory Interpersonal Pleasure Scale (ACIPS), which measured individual differences in the capacity to enjoy interpersonal interactions (Chan et al., 2016). The inventory consists of 17 items, each answered on a 6-point Likert scale from 1 (very false for me) to 6 (very true for me), yielding a total score range of 17–102. Lower total scores reflect a greater likelihood of trait social anhedonia. The Cronbach’s α for the ACIPS in the current study was 0.91 (95% CI [0.90, 0.91]), and McDonald’s ω was 0.91 (95% CI [0.90, 0.92]).

#### 2.2.5 Guilt

The Volitional Action Questionnaire was developed by Zahn and Green to capture aspects of dutifulness and guilt (Jaeckle et al., 2017). The inventory contains eight items, each answered on a 7-point Likert scale from 1 (not at all) to 7 (extremely often), ranging from a total score range of 8–56. Scores on frequency, intensity, and importance questions are summed to guilt and enjoyment. In the current study, this scale was used only for the non-clinical sample, and the Cronbach’s α was 0.78 (95% CI [0.76, 0.79]), and McDonald’s ω was 0.77 (95% CI [0.75, 0.79]).

#### 2.2.6 Depressive symptoms

The Chinese version of the Beck Depression Inventory (BDI-I) was used to measure depressive symptoms during the past 2 weeks (Zheng et al., 1988). The inventory includes 21 items, each answered on a 4-point Likert scale, with higher scores representing a higher occurrence of symptoms associated with depression. Following previous research (Loas et al., 2016), four anhedonia-related items (the anhedonia subscale; items 4, 12, 15, and 21) were removed to avoid construct overlap with the SLIPS. The remaining 17 items produced a total score range from 0 to 51. The Cronbach’s α for BDI-17 in the present study was 0.92 (95% CI [0.91, 0.92]), and McDonald’s ω was 0.92 (95% CI [0.91, 0.93]).

#### 2.2.7 Suicidal ideation

The Chinese version of the Beck Scale for Suicide Ideation (BSS) was used to measure the severity of actual suicidal wishes and plans (Li et al., 2011). The BSS consists of 19 items, each rated on a 3-point Likert scale. Higher total scores reflect stronger suicidal ideation, with a score range of 0–38. In the current study, the first five items were used as a screener measuring suicidal ideation, and the Cronbach’s α was 0.84 (95% CI [0.82, 0.85]) and McDonald’s ω was 0.84 (95% CI [0.82, 0.86]).

#### 2.2.8 Schizotypal personality

The Schizotypal Personality Questionnaire (SPQ) was used to measure schizotypal personality disorder in the general population (Ma et al., 2010). The SPQ is a 74-item self-report inventory comprising three subscales: positive symptoms, negative symptoms, and disorganization, rated dichotomously as “yes” or “no.” Higher scores indicate greater schizotypal personality features. In the current study, this scale was used only for the non-clinical sample, and the Cronbach’s α was 0.96 (95% CI [0.95, 0.96]), and McDonald’s ω was 0.96 (95% CI [0.95, 0.96]).

### 2.3 Procedure

For the non-clinical sample, the participants were recruited via social media platforms (WeChat). The survey was hosted on “Wen Juan Xing” (an online survey platform). Before the survey, all participants provided electronic informed consent. To minimize potential recruitment and self-selection biases, we used strict inclusion criteria and embedded attention-check items within the survey to ensure data quality. All the questionnaires were presented in a fixed order, with the SLIPS administered prior to other measures. For the clinical sample, recruitment was conducted on a one-to-one basis in the hospital. Participants provided written informed consent after a detailed explanation of the study procedures. They were also advised to stop and seek help if they experienced negative emotions at any time, and they were given contact details for mental health services. Participants were compensated with 20 yuan (approximately $2.8) and 1 quality expansion. The ethics board of the Affiliated Mental Health Center, East China Normal University (M202226) approved this project.

### 2.4 Statistical analysis

To rigorously examine the factor structure of the SLIPS, we adopted a split-sample cross-validation approach using the non-clinical sample (*n* = 1,468). The sample was randomly divided into two independent halves. An exploratory factor analysis (EFA) was conducted with the first subsample (n_1_ = 734) using the psych package in R 4.5.1 (R Studio 2025.05.1). Given the ordinal nature of the Likert-type data, polychoric correlations were used instead of Pearson’s correlations. The number of factors to retain was assessed using parallel analysis (PA) (Horn, 1965), the minimum average partial (MAP) test (Velicer, 1976), and the scree plot (Cattell, 1966). The factor structure derived from the EFA was then validated using confirmatory factor analysis (CFA) on the second subsample (n_2_ = 734) using the Lavaan package in R. The weighted least squares mean and variance adjusted (WLSMV) estimator was utilized, which provides robust parameter estimates and standard errors for non-normal, categorical data. Model fit was evaluated using the comparative fit index (CFI), Tucker–Lewis index (TLI), and root mean square error of approximation (RMSEA). As suggested by Hu and Bentler (1999), CFI/TLI ≥ 0.90 and RMSEA ≤0.06 indicate a good fit. Based on these criteria, the original model was revised by examining modification indices, which indicated that residuals from some items were correlated, and by including these partially correlated errors to improve the model fit indices.

Cronbach’s *α* and McDonald’s *ω* coefficient were used to assess the internal consistency, and the intra-class correlation was used to assess the test–retest reliability. Convergent and discriminant validity were assessed using Spearman’s Rho correlations. A two-step hierarchical regression was used to assess incremental validity with depressive symptoms (BDI-17) and suicidal ideation as the outcome variable. Individual differences were examined using the Mann–Whitney U-test due to violations of normality assumptions for the data. Linear mixed models (LMMs) were performed using the lme4 package in R to examine the potential impact of pandemic stress and the predictive effect of the SLIPS on depressive symptoms and suicidal ideation. All continuous predictors were grand-mean centered prior to analysis, and the models were estimated using restricted maximum likelihood (REML). The fixed effects included time, gender, SLIPS, state, and social and trait anhedonia, with depressive symptoms included as a covariate solely in the model for predicting suicidal ideation. A random intercept for subjects was also incorporated into the models.

## 3 Results

### 3.1 Sample characteristics

Kolmogorov–Smirnov tests indicated that the data did not meet the assumption of normality (*p*s<0.05), necessitating the use of non-parametric tests for group comparisons. Compared to the pre-lockdown sample, the age was higher, and there were more women in the during-lockdown sample. No significant differences were found between the SLIPS, other types of anhedonia, depressive symptoms, and suicidal ideation (Table 1). A histogram of the SLIPS score distribution for the non-clinical sample is shown in Supplementary Figure 1. For the clinical sample, no significant differences were found in age, gender, or education. Compared to healthy controls, psychiatric patients showed higher state, social, and trait consummatory anhedonia, depressive symptoms, and suicidal ideation. However, no differences were found in the SLIPS, trait anticipatory, and consummatory anhedonia. Gender differences between the SLIPS and the SHAPS in each sample were also examined, as presented in Supplementary Table 2.

### 3.2 Exploratory factor analysis

In the first subsample, the Scree test and MAP identified a one-factor structure (see Supplementary Figure 2), although the PA indicated an eight-factor solution, which might result from its sensitivity to trivial variance in large samples. Accordingly, a one-factor solution was retained. The first factor accounted for 45.1% of the total variance. The factor loadings of all 23 items ranged from 0.564 to 0.773, and the detailed results are shown in Table 2.

**Table 2:** Factor loadings, standard errors and error variances.

Items	EFA	CFA			
		Unstd. est.	SE	Std. est.	Error var.
1	0.569	1.000	0.067	0.598	0.642
2	0.711	1.198	0.083	0.676	0.486
3	0.569	1.130	0.083	0.812	0.543
4	0.725	1.357	0.094	0.702	0.340
5	0.607	1.173	0.085	0.555	0.507
6	0.679	0.928	0.107	0.737	0.691
7	0.738	1.232	0.091	0.619	0.457
8	0.609	1.049	0.083	0.689	0.606
9	0.605	1.035	0.093	0.649	0.617
10	0.608	1.084	0.091	0.789	0.579
11	0.706	1.157	0.092	0.553	0.520
12	0.773	1.319	0.093	0.825	0.377
13	0.603	0.924	0.088	0.716	0.694
14	0.749	1.215	0.086	0.761	0.472
15	0.790	1.378	0.090	0.827	0.320
16	0.765	1.272	0.089	0.704	0.421
17	0.724	1.196	0.095	0.781	0.488
18	0.631	1.010	0.094	0.604	0.635
19	0.732	1.306	0.091	0.781	0.390
20	0.564	0.877	0.096	0.525	0.725
21	0.660	0.986	0.085	0.590	0.652
22	0.644	1.151	0.091	0.689	0.526
23	0.598	1.156	0.093	0.691	0.522

EFA = Exploratory Factor Analysis (n_1_ = 734); CFA = Confirmatory Factor Analysis (n_2_ = 734); Unstd. est. = Unstandardized Estimate; SE = Standard Error; Std. est. = Standardized Estimate. All factor loadings were statistically significant (*p* < 0.001). The CFA model included 3 correlated residuals to account for local dependence: Items 1&2 (r = 0.55), Items 20&21 (r = 0.35) and Items 22&23 (r = 0.37).

### 3.3 Confirmatory factor analysis

The unidimensional structure was tested in the second subsample using CFA with the WLSMV estimator. Based on modification indices, residual correlations were allowed for three pairs of adjacent items (items 1–2, 20–21, and 22–23). The final model demonstrated satisfactory fit: *χ*^2^(227) = 375.771 (*p* < 0.001), scaled CFI = 0.981, scaled TLI = 0.979, scaled RMSEA = 0.032 (90% CI [0.026, 0.038]), SRMR = 0.055. The factor loadings, standard errors, error variances, and residual correlations are shown in Table 2.

### 3.4 Reliability and validity analysis

The Cronbach’s *α* of the SLIPS was 0.90 (95%CI[0.90, 0.91]), and the McDonald’s *ω* coefficient was 0.91 (95%CI[0.90, 0.92]). Specifically, the Cronbach’s alpha for the pre-lockdown and during-lockdown samples were 0.89 and 0.91, respectively. Test–retest reliability was assessed in the follow-up sample (*n* = 321). The follow-up survey was distributed approximately 1 month after the initial assessment. The intraclass correlation coefficient (ICC; two-way mixed model, absolute agreement, single measure) was 0.55 (95%CI[0.47, 0.62]), indicating moderate stability over the interval. The SLIPS was significantly correlated with other types of anhedonia, depressive symptoms, and suicidal ideation but did not correlate with guilt, see Table 3. The correlations between age and years of education with the SLIPS and SHAPS were also examined in two samples, as presented in Supplementary Table 3. Hierarchical regression analyses (Table 4) showed that after accounting for trait anhedonia, the SLIPS and state anhedonia were independently and strongly associated with depressive symptoms. Crucially, regarding suicidal ideation, the SLIPS was uniquely associated with suicidal ideation while accounting for trait anhedonia.

**Table 3:** Correlations between the SLIPS and other relevant measures in two samples.

Measures	Non-clinical sample								Clinical sample							
	1	2	3	4	5	6	7	8	1	2	3	4	5	6	7	8
SLIPS	-								-							
SHAPS	0.33^*^	-							0.25^*^	-						
ACIPS	−0.43^*^	−0.55^*^	-						−0.44^*^	−0.32^*^	-					
TEPS_ANT	−0.30^*^	−0.48^*^	0.64^*^	-					−0.14	−0.21	0.53^*^	-				
TEPS_CON	−0.23^*^	−0.53^*^	0.54^*^	0.67^*^	-				−0.18	−0.42^*^	0.55^*^	0.78^*^	-			
BDI	0.58^*^	0.31^*^	−0.32^*^	−0.21^*^	−0.23^*^	-			0.52^*^	0.09	−0.32^*^	−0.10	−0.16	-		
SI	0.39^*^	0.25^*^	−0.30^*^	−0.18^*^	−0.16^*^	0.54^*^	-		0.09	0.17	−0.30^*^	−0.08	−0.17	0.35^*^	-	
Guilt	0.03	−0.04	0.07	0.07	0.07	0.14^*^	0.07	-	-	-	-	-	-	-	-	-

SLIPS = Specific Loss of Interest and Pleasure Scale; SHAPS = Snaith-Hamilton Pleasure Scale; ACIPS = Anticipatory and Consummatory Interpersonal Scale; TEPS-ANT = Anticipatory anhedonia in Temporal Experience of Pleasure Scale; TEPS-CON = Consummatory anhedonia in Temporal Experience of Pleasure Scale; BDI = 17 items of Beck Depression Inventory; SI = Suicidal Ideation. Bonferronia correction, ^*^p < 0.05.

**Table 4:** Hierarchical multiple regression analyses.

Samples	Dependent variables	Predictors	Step 1				Step 2			
			*B*(*SE*)	95%CI for B	*β*	*p*	*B*(*SE*)	95%CI for B	*β*	*p*
Non-clinical sample	Depressive symptoms	TEPS-ANT	0.05 (0.05)	[−0.05, 0.14]	0.04	0.332	0.10 (0.04)	[0.02, 0.18]	0.08	0.019
		TEPS-CON	−0.07 (0.04)	[−0.15, 0.00]	−0.07	0.052	−0.07 (0.03)	[−0.14, −0.00]	−0.07	0.042
		ACIPS	−0.22 (0.02)	[−0.27, −0.18]	−0.36	<0.001	−0.05 (0.02)	[−0.09, −0.01]	−0.08	0.015
		SHAPS					0.20 (0.04)	[0.12, 0.28]	0.14	<0.001
		SLIPS					0.58 (0.03)	[0.53, 0.64]	0.50	<0.001
		R^2^	0.144				0.371			
		ΔF	69.82^***^				225.10^***^			
	Suicidal ideation	TEPS-ANT	−0.02 (0.01)	[−0.04, 0.00]	−0.06	0.047	−0.02 (0.01)	[−0.03, 0.00]	−0.06	0.075
		TEPS-CON	0.02 (0.01)	[0.01, 0.04]	0.09	0.003	0.02 (0.01)	[0.01, 0.04]	0.09	0.003
		ACIPS	−0.02 (0.00)	[−0.03, −0.02]	−0.16	<0.001	−0.02 (0.00)	[−0.03, −0.01]	−0.14	<0.001
		BDI	0.14 (0.01)	[0.13, 0.15]	0.60	<0.001	0.13 (0.01)	[0.12, 0.15]	0.57	<0.001
		SHAPS					0.01 (0.01)	[−0.01, 0.03]	0.03	0.246
		SLIPS					0.02 (0.01)	[0.00, 0.03]	0.06	0.040
		R^2^	0.460				0.463			
		ΔF	265.71^***^				3.02^*^			
Clinical sample	Depressive symptoms	TEPS-ANT	0.31 (0.13)	[0.05, 0.57]	0.31	0.018	0.19 (0.13)	[−0.06, 0.44]	0.19	0.133
		TEPS-CON	−0.14 (0.12)	[−0.37, 0.10]	−0.15	0.255	−0.10 (0.12)	[−0.33, 0.13]	−0.11	0.392
		ACIPS	−0.18 (0.05)	[−0.27, −0.09]	−0.34	<0.001	−0.09 (0.05)	[−0.18, 0.00]	−0.17	0.062
		SHAPS					−0.02 (0.12)	[−0.25, 0.21]	−0.01	0.864
		SLIPS					0.36 (0.07)	[0.23, 0.50]	0.39	<0.001
		R^2^	0.097				0.222			
		ΔF	6.63^***^				14.79^***^			
	Suicidal ideation	TEPS-ANT	0.03 (0.03)	[−0.03, 0.09]	0.13	0.286	0.03 (0.03)	[−0.03, 0.10]	0.14	0.272
		TEPS-CON	−0.03 (0.03)	[−0.08, 0.03]	−0.11	0.358	−0.02 (0.03)	[−0.07, 0.04]	−0.08	0.544
		ACIPS	−0.03 (0.01)	[−0.05, −0.01]	−0.22	0.008	−0.04 (0.01)	[−0.06, −0.01]	−0.28	0.002
		BDI	0.09 (0.02)	[0.06, 0.13]	0.37	<0.001	0.11 (0.02)	[0.08, 0.15]	0.44	<0.001
		SHAPS					0.04 (0.03)	[−0.02, 0.09]	0.09	0.185
		SLIPS					−0.05 (0.02)	[−0.08, −0.01]	−0.20	0.008
		R^2^	0.239				0.271			
		ΔF	14.54^***^				4.01^*^			

TEPS-ANT = Anticipatory anhedonia in Temporal Experience of Pleasure Scale; TEPS-CON = Consummatory anhedonia in Temporal Experience of Pleasure Scale; ACIPS = Anticipatory and Consummatory Interpersonal Scale; SHAPS = Snaith-Hamilton Pleasure Scale; SLIPS = Specific Loss of Interest and Pleasure Scale; BDI = 17 items of Beck Depression Inventory. The regression coefficients were standardized. Collinearity diagnostics indicated no multicollinearity issues (Maximum VIF = 4.05). ^*^p < 0.05, ^***^p < 0.001.

### 3.5 Individual differences in changes in anhedonia

Compared to the low BDI group (total BDI score < 16, M ± SD: 4.99 ± 5.05, *n* = 924), the high group (total BDI score ≥ 16, M ± SD: 12.88 ± 8.77, *n* = 327) showed significantly higher SLIPS scores (*t*_(405)_ = −15.41, *p* < 0.001, *d* = 1.10). Students ranking in the top 10% (scores > 46) and bottom 10% (scores < 5) of the SPQ were divided into high/ low schizotypal groups. The high schizotypal group (12.87 ± 9.21) reported higher SLIPS scores than the low group (1.99 ± 3.49) (*t*_(174)_ = −12.77, *p* < 0.001, *d* = 1.56). However, no gender difference in the SLIPS was found (*t*_(1249)_ = 0.11, *p* = 0.910, *d* = 0.01).

### 3.6 Effects of the COVID-19 pandemic on the SLIPS

The characteristics of the longitudinal sample across the pandemic are presented in Supplementary Table 1. No significant difference in the SLIPS was found between baseline (6.56 ± 6.56) and follow-up (6.56 ± 6.40), considering gender and age as covariates (*F*(1, 318) = 0.961, *p* = 0.328). The results of the longitudinal measurement invariance indicated good model fit at the baseline configural level. Subsequent constraints imposed on metric, scalar, and residual invariance resulted in negligible changes in model fit (ΔCFI < 0.01). These findings support strict measurement invariance across two time points, demonstrating that the factor structure, factor loadings, intercepts, and item residuals of the SLIPS remained stable across repeated measurements (Table 5).

**Table 5:** The model fit indices of measurement invariance (*n* = 321).

Model	χ^2^	df	CFI	ΔCFI	TLI	ΔTLI	SRMR	RMSEA (90% CI)
Configural Invariance	1160.221	959	0.966	-	0.964	-	0.086	0.026 (0.020, 0.031)
Metric Invariance	1164.666	981	0.969	0.003	0.967	0.003	0.091	0.024 (0.018, 0.030)
Scalar Invariance	1180.257	981	0.967	−0.002	0.965	−0.002	0.086	0.025 (0.019, 0.030)
Residual Invariance	1188.159	1,004	0.969	0.002	0.968	0.003	0.091	0.024 (0.018, 0.029)

df = degrees of freedom; CFI = Comparative Fit Index; TLI = Tucker-Lewis Index; SRMR = Standardized Root Mean Squared Residual; RMSEA = Root Mean Square Error of Approximation; CI = Confidence Interval. The degrees of freedom for the scalar invariance model were identical to the metric model due to parameter redundancies in threshold constraints when using WLSMV estimation, but the change in fit indices (CFI) suggests that scalar invariance was supported.

LMMs were conducted to examine the predictors of depressive symptoms and suicidal ideation over time (Table 6). Regarding depressive symptoms, the main effect of time was not significant, while higher SLIPS scores and higher trait consummatory anhedonia could significantly predict greater depressive symptoms. After accounting for the effect of depressive symptoms, higher SLIPS scores, higher trait anticipatory anhedonia, and lower trait consummatory anhedonia exhibited a significant predictive effect on higher suicidal ideation.

**Table 6:** Multivariate mixed model examining the predictors of depressive symptoms and suicidal ideation during lockdown (*n* = 321).

Variables	Depressive symptoms				Suicidal ideation			
	Estimate	*t*	*p*	95%CI	Estimate	*t*	*p*	95%CI
BDI	-	-	-	-	0.13	16.71	<0.001	[0.11, 0.14]
Time	−0.26	−0.64	0.524	[−1.07, 0.55]	−0.07	−0.88	0.382	[−0.22, 0.09]
Gender	−1.05	−1.35	0.180	[−2.59, 0.48]	0.23	1.54	0.126	[−0.06, 0.52]
SLIPS	0.43	8.59	<0.001	[0.33, 0.53]	0.02	2.47	0.014	[0.01, 0.04]
SHAPS	0.10	1.86	0.064	[−0.01, 0.21]	0.02	1.79	0.075	[0.00, 0.04]
ACIPS	−0.04	−1.33	0.186	[−0.10, 0.02]	−0.01	−0.90	0.371	[−0.02, 0.01]
TEPS-ANT	0.02	0.25	0.803	[−0.11, 0.14]	−0.03	−2.95	0.003	[−0.06, −0.01]
TEPS-CON	−0.11	−2.13	0.033	[−0.21,-0.01]	0.03	2.93	0.004	[0.01, 0.05]

BDI = 17 items of Beck Depression Inventory; SI = Suicidal Ideation; SLIPS = Specific Loss of Interest and Pleasure Scale; SHAPS = Snaith-Hamilton Pleasure Scale; ACIPS = The Anticipatory and Consummatory Interpersonal Scale; TEPS-ANT = Anticipatory anhedonia in Temporal Experience of Pleasure Scale; TEPS-CON = Consummatory anhedonia in Temporal Experience of Pleasure Scale.

### 3.7 Further validation in a clinical sample

The same unidimensional structure was tested in the clinical sample using CFA with the WLSMV estimator. The final model demonstrated satisfactory fit: *χ*^2^(230) = 358.920 (*p* < 0.001), scaled CFI = 0.984, scaled TLI = 0.983, scaled RMSEA = 0.054 (90% CI [0.043, 0.065]), SRMR = 0.069. The Cronbach’s *α* was 0.96 (95%CI [0.95, 0.97]), and the McDonald’s *ω* coefficient was 0.96 (95%CI [0.95, 0.97]). The same two-step hierarchical regression found that compared with the state anhedonia, the SLIPS was positively associated with depressive symptoms, whereas the SLIPS showed a significant negative association with suicidal ideation (Table 4).

## 4 Discussion

The current study investigated the psychometric properties of the SLIPS using Chinese non-clinical and clinical samples. The scale demonstrated a unidimensional structure, excellent internal consistency, and moderate stability at a 1-month interval. It also showed good convergent and divergent validity. Compared to other types of anhedonia, the SLIPS could be incrementally associated with depressive symptoms and suicidal ideation in non-clinical and clinical populations, suggesting that the SLIPS, which assesses recent changes in anhedonia, is a unique and promising tool for further anhedonia research.

Consistent with the original study (Winer et al., 2014b), CFA results confirmed that all items loaded reasonably on a single factor, supporting the SLIPS as a unidimensional measure for assessing recent changes in anhedonia. The McDonald’s ω coefficient was excellent, while the test–retest coefficient was moderate, which is consistent with its unique element of capturing fluctuations in recent anhedonia. As expected, significant correlations between the SLIPS and other types of anhedonia were found, suggesting that the SLIPS is closely converged with the construct of anhedonia. Moreover, the SLIPS remained significantly associated with more depressive symptoms and higher suicidal ideation after accounting for the shared relationship of trait, social, and state anhedonia, representing strong incremental validity of the SLIPS, which supports the independence of recent changes in anhedonia from the state anhedonia. Consistent with this independence, preliminary analyses indicated a limited association between the SLIPS and guilt; therefore, guilt was not retained as a dependent variable in the current analysis.

The same one-factor structure and excellent internal consistency were further confirmed in psychiatric patients, supporting the utility of the SLIPS in Chinese clinical settings. Regarding incremental validity, the SLIPS remained a significant predictor of both depressive symptoms and suicidal ideation after controlling for other anhedonia measures. Interestingly, the SLIPS positively predicted depressive symptoms, which was the same as the previous finding that recent changes in anhedonia were a specific risk factor for depression (Winer et al., 2014b; Yang et al., 2022). However, the SLIPS showed a negative association with suicidal ideation in the clinical sample, which contrasts with the results in our non-clinical sample and previous studies (Winer et al., 2014a). The discrepancy might represent a statistical suppressor effect (Watson et al., 2013). As the BDI strongly accounts for the distress and hopelessness driving suicidal ideation (*β* = 0.44, *p* < 0.001), the residual variance in the SLIPS might capture a distinct facet of severe anhedonia, such as motivational anergy or profound emotional flattening, which could paradoxically reduce the active energy required for suicidal planning (Loas et al., 2018).

Since our data were collected during the COVID-19 pandemic, the effect of the pandemic on the SLIPS was assessed. First, the establishment of strict measurement invariance confirmed that the factor structure of the SLIPS remained stable across the pandemic, indicating higher generalizability across different social environments (Wellan et al., 2021). Second, no significant main effect of time on depressive symptoms or suicidal ideation was found, suggesting that symptom levels remained stable during the early lockdown phase, which aligns with a prior finding that state anhedonia was stable during lockdown (Wellan et al., 2021). Importantly, the LMM results were largely consistent with the cross-sectional findings and offered stronger predictive evidence. Specifically, the SLIPS significantly predicted both depressive symptoms and suicidal ideation over time. This is consistent with previous studies showing that higher anhedonia could predict severe depressive symptoms during the pandemic (Moccia et al., 2020) and that recent changes in anhedonia were a risk factor for current suicidal ideation (Dosogne et al., 2022). These findings support that the SLIPS, representing the construct of recent changes in anhedonia, could accurately predict future depression and suicide risk within the unstable context of the pandemic. It is worth noting that although previous research has reported fluctuations in guilt during the lockdown (Paleari et al., 2025), the current study prioritized understanding how anhedonia contributed to variations in depressive symptoms and suicidal ideation. Consequently, as guilt was conceptually distinct from anhedonia, it was considered beyond the scope of the present longitudinal investigation.

Several limitations must be noted. First, our non-clinical sample was recruited via non-probability convenience sampling and was predominantly female, thereby limiting the generalizability of our findings. Despite no gender difference in the SLIPS, further replication with more representative sampling strategies was needed, along with tests for measurement invariance across gender and age to mitigate potential bias. Second, although a transdiagnostic psychiatric group was included to capture the transdiagnostic feature of anhedonia (Gandhi et al., 2022; Whitton and Pizzagalli, 2022), the sample sizes for specific diagnostic subgroups (e.g., MDD) were insufficient for reliable disorder-specific comparisons. Future research with larger clinical samples is essential to examine potential diagnostic heterogeneity in the SLIPS. Third, self-report measures were subject to methodological constraints, which might introduce common-method bias. Future psychometric evaluations should incorporate advanced techniques [Item Response Theory or Differential Item Functioning (Hambleton and Jones, 1993)] to further enhance the scale’s precision at different trait levels. Additionally, potential floor effects in non-clinical populations and the impact of item recoding strategies on score distribution should be considered in future applications. Furthermore, considering the lockdown as acute stress, the influence of chronic stress and early adverse life events on recent changes in anhedonia also warrants further investigation. Finally, although the study was not a cohort or randomized controlled trial, it was not preregistered.

## 5 Conclusion

In summary, the SLIPS showed consistent, satisfactory reliability and validity in both non-clinical and clinical populations, providing promising evidence of its utility in Chinese settings. However, further validation is needed in independent clinical subgroups and diverse cultural contexts. Overall, the SLIPS appears to be a valuable tool for assessing recent changes in anhedonia.

## Data Availability

The raw data supporting the conclusions of this article will be made available by the authors, without undue reservation.
