# Supplementary material for: Validation of the specific loss of interest and pleasure scale in non-clinical and clinical populations
Source: Front Psychol. 2026 Mar 24;17:1706503. doi: 10.3389/fpsyg.2026.1706503 (PMC13055622; doi:10.3389/fpsyg.2026.1706503)
Supplement: Supplementary file 1 [file supplementary_file_1.docx]

## Supplementary Text 1

The characteristics of the longitudinal sample are reported in Table 1. Compared with pre-lockdown, participants showed higher trait anticipatory and consummatory anhedonia, lower depressive symptoms and guilt during lockdown. However, no significant differences were found in the SLIPS, state and social anhedonia or suicidal ideation.

## Table 1 - Characteristics of longitudinal sample (n=321)

| Variable | Pre-lockdown(T1) | During-lockdown(T2) | *z* | *p* |
| --- | --- | --- | --- | --- |
| SLIPS | 6.56±6.56 | 6.56±6.40 | 0.01 | .991 |
| SHAPS | 23.19±6.22 | 23.44±6.30 | 0.28 | .780 |
| TEPS-ANT | 37.56±7.31 | 38.45±6.76 | 2.42 | .015 |
| TEPS-CON | 44.19±8.13 | 44.95±8.30 | 2.03 | .042 |
| ACIPS | 75.93±13.07 | 75.08±13.27 | 1.24 | .214 |
| Guilt | 18.09±5.15 | 17.37±5.00 | 2.33 | .020 |
| BDI | 7.92±8.20 | 7.64±8.68 | 2.10 | .036 |
| SI | 1.13±1.95 | 1.03±1.85 | 1.33 | .183 |

SLIPS = Specific Loss of Interest and Pleasure Scale; SHAPS = Snaith-Hamilton Pleasure Scale; TEPS-ANT = Anticipatory anhedonia in Temporal Experience of Pleasure Scale; TEPS-CON = Consummatory anhedonia in Temporal Experience of Pleasure Scale; ACIPS = The Anticipatory and Consummatory Interpersonal Scale; BDI = 17 items of Beck Depression Inventory; SI = Suicidal Ideation; Data are presented as n or M±SD.

## Supplementary Text 2

Gender differences in SLIPS and SHAPS in each sample were examined using the Mann-Whitney U test. Males exhibited higher state anhedonia than females in the non-clinical sample.

## Table 2 - Gender differences in two samples

|  |  | **Non-clinical sample** | | | | **Clinical Sample** | | | |
| --- | --- | --- | --- | --- | --- | --- | --- | --- | --- |
| Variable | | Female  (n=905) | Male  (n=346) | *z* | *p* | Female  (n=141) | Male  (n=83) | *z* | *p* |
| SLIPS | | 7.04±6.91 | 7.09±7.71 | -0.91 | .361 | 8.43±11.04 | 5.87±7.40 | 0.12 | .905 |
| SHAPS | | 22.91±5.61 | 24.53±6.76 | 4.17 | <.001 | 23.52±5.76 | 23.42±6.32 | -1.45 | .148 |

*Notes*: SLIPS = Specific Loss of Interest and Pleasure Scale; SHAPS = Snaith-Hamilton Pleasure Scale

## Supplementary Text 3

Spearman rank correlations were conducted to examine the relationship of age and years of education with SLIPS and SHAPS. Longer years of education was associated with lower state anhedonia in the non-clinical sample.

## Table 3 - Spearman Rank Correlations in two samples

|  | **Non-clinical Sample** | | | | **Clinical Sample** | | | |
| --- | --- | --- | --- | --- | --- | --- | --- | --- |
|  | 1 | 2 | 3 | 4 | 1 | 2 | 3 | 4 |
| Age | - |  |  |  | - |  |  |  |
| Education (years) | .17^**^ | - |  |  | -.44^**^ | - |  |  |
| SLIPS | .02 | -.05 | - |  | -.01 | .09 | - |  |
| SHAPS | .03 | -.12^*^ | .33^*^ | - | .17^*^ | -.11 | .25^**^ | - |

*Notes*: SLIPS = Specific Loss of Interest and Pleasure Scale; SHAPS = Snaith-Hamilton Pleasure Scale, ^*^*p*<.05, ^**^*p*<.01

## Supplementary Figure 1 - Histogram of SLIPS score distribution


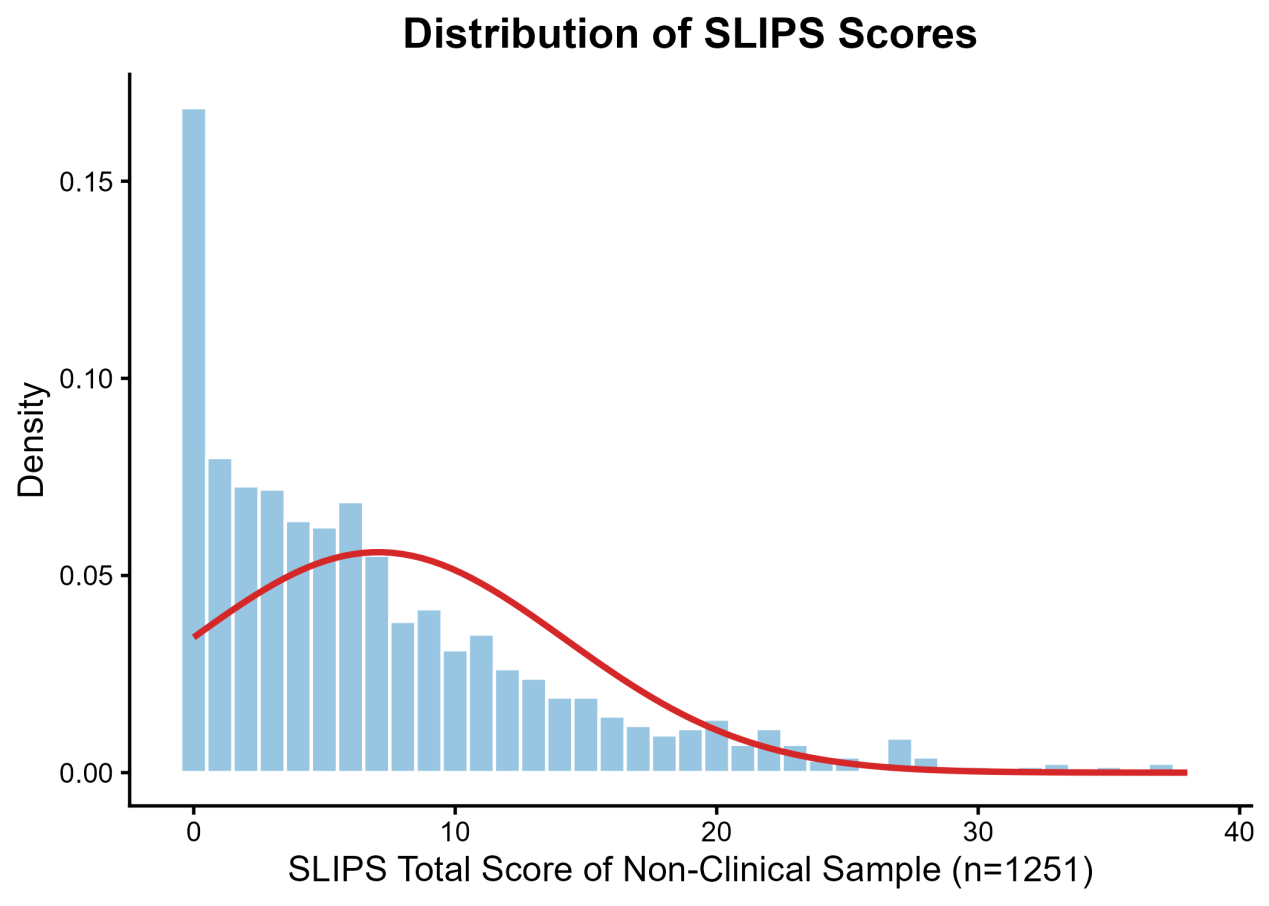


## Supplementary Figure 2 - Scree plot with Parallel Analysis


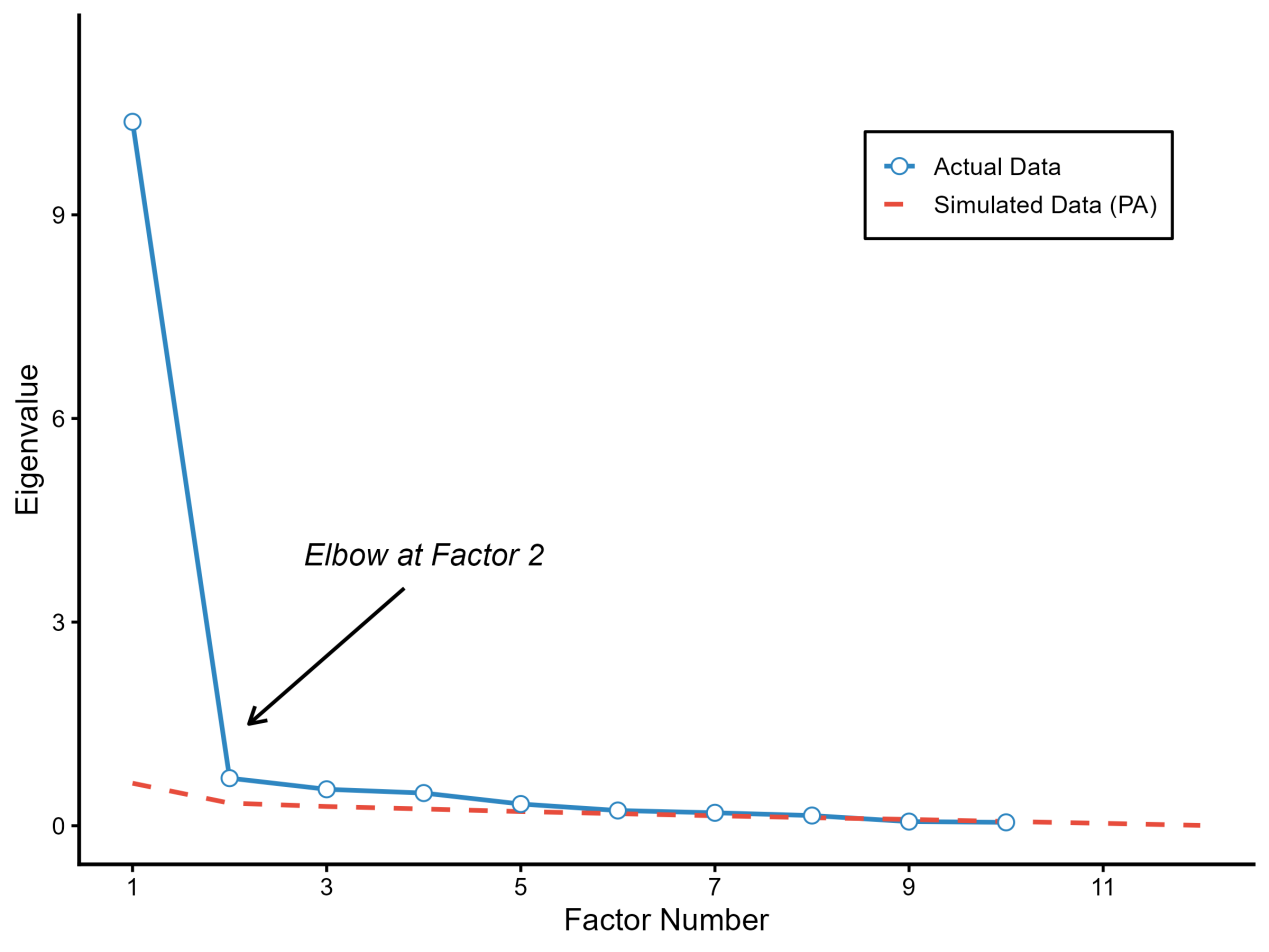


**Appendix A**

The Chinese SLIPS items are as follows:

我仍然喜欢和朋友出去。

我不像以前那样喜欢和朋友出去。

我不再喜欢和朋友出去。

我从来都不喜欢和别人出去。

我喜欢和朋友在一起。

我不像以前那样喜欢和朋友在一起。

我不再喜欢和朋友在一起。

我没有朋友。

我对集体娱乐活动感兴趣。

我对集体娱乐活动的兴趣比以前减少。

我对集体娱乐活动失去大部分兴趣。

我从不喜欢集体娱乐。

有朋友关心我，让我很开心。

有朋友关心我，我不像以前那样开心。

有朋友关心我，不再让我开心。

我没有关心我的朋友。

当人们向我倾诉烦恼时，我通常很感兴趣。

最近，当人们向我倾诉烦恼时，我不像以前那样感兴趣。

最近，当人们向我倾诉烦恼时，我几乎不再感兴趣。

当人们向我倾诉烦恼时，我从来都不感兴趣。

我爱着我的亲人。

我对亲人的爱比以前减少。

我对亲人几乎不再有爱。

我从来没有爱过别人。

我喜欢和朋友长时间的聊天。

我不像以前那样喜欢和朋友长时间的聊天。

我不再喜欢和朋友长时间的聊天。

我从来不喜欢和朋友长时间的聊天

我喜欢结交新朋友。

我不像以前那样喜欢结交新朋友。

我不再有兴趣认识新朋友。

我从来没有兴趣认识新朋友。

我更喜欢与别人一起生活，而不是一个人住。

我不像以前那样喜欢和别人一起生活。

最近，我对与别人一起生活失去大部分的兴趣。

我习惯一个人住，而不是与别人一起生活。

我对别人的日常活动和观点感兴趣。

我不像以前那样对别人的日常活动和观点感兴趣。

我对别人的日常活动和观点不再感兴趣。

我对别人的日常活动和观点从来都不感兴趣。

我喜欢与人建立牢固的关系。

我不像以前那样喜欢与人建立牢固的关系。

我对与人建立牢固的关系失去大部分的兴趣。

我对与人建立牢固的关系从来都不感兴趣。

我喜欢与人交谈。

我不像以前那样喜欢与人交谈。

我几乎再也找不到与人交谈的乐趣。

我从来都不喜欢和人交谈。

我能从体育活动中获得乐趣。

我不像以前那样能从体育活动中获得乐趣。

我几乎不再能从体育活动中获得乐趣。

我从未从体育活动中获得过乐趣。

我对别人没有失去兴趣。

我对别人失去一些兴趣。

我对别人失去大部分的兴趣。

我对别人从来都不感兴趣。

我喜欢我的朋友。

我不像以前那样喜欢我的朋友。

我不再喜欢我的朋友。

我从来都不喜欢我的朋友。

我喜欢与同事或同学交流。

我与同事或同学交流比以前减少。

我几乎不再与同事或同学交流。

我从来都不与同事或同学交流。

朋友们喜欢我。

朋友们不像以前那样喜欢我。

朋友们似乎不再喜欢我。

我从来没有觉得朋友们喜欢我。

我在乎自己的工作表现。

我不像以前那样在乎自己的工作表现。

我不再在乎自己的工作表现。

我从来不在乎自己的工作表现。

我喜欢和别人一起吃饭。

我不像以前那样喜欢和别人一起吃饭。

我不再喜欢和别人一起吃饭。

我从来都不喜欢和别人一起吃饭。

我对喜欢的电影没有失去兴趣。

我不像以前那样对喜欢的电影感兴趣。

我对喜欢的电影几乎不再感兴趣。

我从来都不喜欢看任何类型的电影。

我对自己的爱好没有失去兴趣。

我对自己的爱好的兴趣比以前减少。

我对自己的爱好失去大部分的兴趣。

我一直很难对任何活动产生兴趣。

我期待着生活中会出现一些激动人心的事。

我不像以前那样期待着生活中出现一些激动人心的事。

我不再期待生活中会发生任何事。

我从未期待生活中出现一些激动人心的事。

在盛大活动的前一晚，我通常很兴奋。

在盛大活动的前一晚，我不像以前那样兴奋。

在盛大活动的前一晚，我不再感到兴奋。

在盛大活动的前一晚，我从来都不兴奋。
